# Evaluation of a Web-based Intervention Providing Tailored Advice for Self-management of Minor Respiratory Symptoms: Exploratory Randomized Controlled Trial

**DOI:** 10.2196/jmir.1599

**Published:** 2010-12-15

**Authors:** Lucy Yardley, Judith Joseph, Susan Michie, Mark Weal, Gary Wills, Paul Little

**Affiliations:** ^4^School of MedicineUniversity of SouthamptonSouthamptonUnited Kingdom; ^3^School of Electronics and Computer ScienceUniversity of SouthamptonSouthamptonUnited Kingdom; ^2^Department of Clinical, Educational and Health PsychologyUniversity College LondonLondonUnited Kingdom; ^1^School of PsychologyUniversity of SouthamptonSouthamptonUnited Kingdom

**Keywords:** Internet, self-care, consumer health information, communication, common cold, influenza

## Abstract

**Background:**

There has been relatively little research on the role of web-based support for self-care in the management of minor, acute symptoms, in contrast to the wealth of recent research into Internet interventions to support self-management of long-term conditions.

**Objective:**

This study was designed as an evaluation of the usage and effects of the “Internet Doctor” website providing tailored advice on self-management of minor respiratory symptoms (eg, cough, sore throat, fever, runny nose), in preparation for a definitive trial of clinical effectiveness. The first aim was to evaluate the effects of using the Internet Doctor webpages on patient enablement and use of health services, to test whether the tailored, theory-based advice provided by the Internet Doctor was superior to providing a static webpage providing the best existing patient information (the control condition). The second aim was to gain an understanding of the processes that might mediate any change in intentions to consult the doctor, by comparing changes in relevant beliefs and illness perceptions in the intervention and control groups, and by analyzing usage of the Internet Doctor webpages and predictors of intention change.

**Methods:**

Participants (N = 714) completed baseline measures of beliefs about their symptoms and self-care online, and were then automatically randomized to the Internet Doctor or control group. These measures were completed again by 332 participants after 48 hours. Four weeks later, 214 participants completed measures of enablement and health service use.

**Results:**

The Internet Doctor resulted in higher levels of satisfaction than the control information (mean 6.58 and 5.86, respectively; *P* = .002) and resulted in higher levels of enablement a month later (median 3 and 2, respectively; *P* = .03). Understanding of illness improved in the 48 hours following use of the Internet Doctor webpages, whereas it did not improve in the control group (mean change from baseline 0.21 and -0.06, respectively, *P* = .05). Decline in intentions to consult the doctor between baseline and follow-up was predicted by age (beta = .10, *P*= .003), believing before accessing the website that consultation was necessary for recovery (beta = .19, *P* < .001), poor understanding of illness (beta = .11, *P* = .004), emotional reactions to illness (beta = .15, *P* <.001), and use of the Diagnostic section of the Internet Doctor website (beta = .09, *P* = .007).

**Conclusions:**

Our findings provide initial evidence that tailored web-based advice could help patients self-manage minor symptoms to a greater extent. These findings constitute a sound foundation and rationale for future research. In particular, our study provides the evidence required to justify carrying out much larger trials in representative population samples comparing tailored web-based advice with routine care, to obtain a definitive evaluation of the impact on self-management and health service use.

## Introduction

There has been relatively little research on the role that web-based support for self-management might play in the management of minor, acute symptoms, in contrast to the wealth of recent research into Internet interventions to support self-management of long-term conditions. It is well known that patients already self-care for the vast majority of minor symptoms, making their own decisions about whether and how to manage symptoms themselves (eg, using over-the-counter remedies) or whether to seek medical advice [1]. Nevertheless, over half the population in the United Kingdom consult their doctor each year for a minor symptom, and acute respiratory symptoms (eg, cough, sore throat) are the most common cause of consultation [2,3]. Only a tiny minority of the general public use the Internet for routine health care activities such as contacting their own doctor [4].

There are compelling reasons for finding ways to use the Internet to support patients to self-manage minor symptoms. Most people say that they would find it convenient and empowering to be given enough information to be able to self-manage without seeing their doctor [5-7]. Policy makers and clinicians are concerned that unnecessary consultations are an inefficient use of scarce health care resources [8,9]. However, there are also significant barriers to using the Internet for self-care. Both patients and doctors are concerned about the quality of information provided, and whether patients have the necessary skills and confidence to evaluate and manage their symptoms [3,10-12].

Prior to the advent of mass Internet access, patient education about self-management of minor symptoms was attempted by means of booklets and other media with some degree of success [13-16], although effects on consultation rates were typically very modest. A plausible advantage of using the Internet as a means of providing advice about self-management is that it can be tailored to symptoms, and should therefore be, and be perceived as, more personally relevant and hence accurate [17]. Qualitative evaluations of websites that provide tailored information for self-diagnosis and self-management of symptoms [18,19] suggest that they are seen as a useful complement to medical advice, but that it can be difficult to provide patients with advice that is sufficiently personalized, accessible, and detailed to replace consultation. However, the assumption that tailoring advice to the individual’s symptoms will improve patient satisfaction and outcomes has not yet been experimentally tested in the context of web-based advice for self-management of common symptoms.

Previous studies of providing information on self-management of symptoms have been largely pragmatic, focusing simply on whether providing educational materials leads to better outcomes than routine care. For example, an observational study of providing a student population with online digital triage advice on whether they needed to seek medical care for minor symptoms was able to demonstrate satisfactory uptake and excellent concordance between the online advice and clinical diagnoses [20]. However, if Internet-delivered care is to become a widely accepted and well-integrated part of efficient routine health care, then we need to understand better how and why it might be welcomed and used effectively [14]. Theory-based psychological explanations of how people decide whether they can self-manage symptoms may help us to understand how interventions can be designed to better support self-care.

According to the Social Cognitive Theory, performance of any behavior is typically predicted by confidence that one can carry out the behavior successfully (self-efficacy) and beliefs about the likely consequences (“outcome expectancies”) [21-23]. Thus, advice on how to self-manage symptoms and evidence that the advice has worked for others should improve confidence in the ability to self-care, while reassurance that symptoms are not indicative of serious illness requiring medical care should reduce beliefs that consultation is necessary for recovery. In addition, the Common Sense Model of Self-regulation of health and illness [24] highlights perceptions of illness that are likely to affect self-management of symptoms, such as whether the symptoms cause emotional reactions or are not well understood [25]; providing information about these aspects of symptoms may provide reassurance and reduce the need to consult the doctor. Finally, the Theory of Planned Behavior [26] proposes that the effects of beliefs on behavior are mediated by conscious intentions. A small observational study confirmed that intention to comply with the advice provided by a web-based system providing tailored advice for common symptoms was a strong predictor of reported compliance with the advice 3 months later [27].

This study forms part of a program of research into how theory and evidence can be used to design an intervention that will help patients to self-manage minor respiratory symptoms without seeking medical help. In accordance with best practice in the development of complex interventions [28], it was designed as an exploratory or phase 2 randomized controlled trial (RCT) that would provide an initial evaluation of the usage and effects of the “Internet Doctor” website. The first aim of the study was to evaluate the effects of using the Internet Doctor webpages on the target outcomes for the main trial, namely patient “enablement” [29] (ie, perceived ability to self-manage health and illness) and use of health services (ie, contacting the doctor or other health care services). The control condition was a webpage consisting of advice previously shown to be effective in reducing consultations and improving patient confidence to self-care [9]. This design provides a direct test of whether tailored, theory-based advice is more effective than the best existing information and advice. The second aim was to gain an understanding of the processes that might mediate any change in intentions to consult the doctor, by comparing changes in consultation intentions and in relevant beliefs and illness perceptions in the intervention and control groups, and levels of satisfaction with the website advice. The third aim was to examine whether outcomes were predicted, as expected, by beliefs about self-care and illness perceptions, and use of our theory-based advice. This was addressed by analyzing usage of the Internet Doctor webpages and predictors of change in consultation intentions.

## Methods

### Design and Procedure

This study was designed as an exploratory or phase-2 RCT [28] in preparation for a definitive trial of clinical effectiveness. As such, it has some but not all the characteristics required for a definitive trial. Participants were automatically assigned to the intervention and control groups and were blind to group assignment. However, the trial was not registered, and no sample size calculation was possible or necessary, since an aim of the study was to provide data from which required sample size for a definitive trial could be calculated and the study was not powered as a definitive test of intervention effects. Moreover, our participants were online volunteers with unknown characteristics who could not be followed up rigorously, which precluded meaningful intention-to-treat analysis, whereas a definitive trial would require a clinical sample with known baseline characteristics that could be followed up comprehensively and objectively through their medical records.

The study was approved by the ethics committee of the School of Psychology, University of Southampton. Participants were recruited between October 2009 and March 2010 (the UK winter respiratory infection season) by advertisements providing the website uniform resource locator for the intervention and inviting adults with cold or flu symptoms to try out the website. We specifically targeted university students, as our own qualitative research [30] had suggested that young people with little experience of self-managing minor symptoms on their own were more likely to need and benefit from advice. Advertisements were sent by email to students in 55 university departments in the United Kingdom, distributed as posters and flyers around three university campuses, and placed on websites and at other public locations. Participants who logged onto the website first gave informed consent online (to give their views on one of two versions of self-management advice) and completed the baseline questionnaire. They were then automatically randomized to the intervention (Internet Doctor) or control group by the web-based software, but were not informed which group they were in. The control group was provided with precisely the same advice as that given in the previous successful trial of booklet-based self-care information [9], delivered as a static webpage. The intervention webpages are described below.

Participants were sent an automatic email invitation to complete the intermediate follow-up 48 hours after accessing the intervention, and an invitation to complete the final follow-up after 4 weeks. An incentive (being entered into a prize draw for £100) was offered for completion of the follow-up measures, and nonrespondents received up to two additional reminders to complete the follow-up.

### The Internet Doctor Intervention

**Figure 1:**
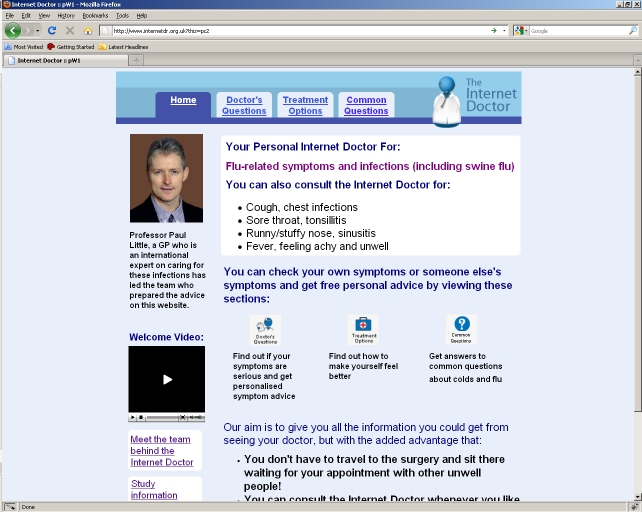
Homepage of the Internet Doctor website

The intervention was a fully automated digital triage system that provided tailored computer-generated advice. Participants were presented with a homepage (Figure 1) explaining what the site offered, with links to details about the medical expert on the team (PL) and the medical evidence the advice was based on. From this homepage participants could choose to access Diagnostic pages, Treatment pages providing self-management information, or Common Questions (see Multimedia Appendix 1 for illustrative screenshots of all of these sections).

The Diagnostic pages first asked a series of questions about the participant’s symptoms; participants completed these pages for one symptom at a time, and could choose from cough, sore throat, fever, and runny/stuffy nose. Then a complex algorithm provided appropriate tailored advice on whether they needed to contact health services for that symptom (see Table 1). There were options to click on the answers to further questions about their symptoms and possible diagnoses. Participants who selected the Treatment pages could then choose between information about natural remedies or over-the-counter medication for symptoms, and advice on how to boost their immune system. The Common Questions section addressed common concerns and misconceptions about symptoms and treatment.

**Table 1:** Varieties of advice provided by the Internet Doctor^a^

Advice type	Symptom reports prompting this advice
Contact NHS Direct^b^ immediately and then your doctor (gives list of symptoms reported that led to this advice)	Symptoms indicating a serious, acute condition (eg, meningitis or septicemia)
You should contact NHS Direct (gives list of symptoms reported that led to this advice)	Symptoms lasting and/or moderately severe (eg, fever ≥38.5^o^ for ≥3 days, cough for ≥4 weeks, breathing getting worse) OR less severe symptoms together with other risk factors (eg, older age, chronic conditions, immune system suppression)
Your symptoms are not a sign of serious illness and you do not need to contact the doctor at present (gives reassuring explanation of symptoms and advises to reconsult website if symptoms persist or worsen)	Symptoms acute and not severe or worsening

^a^ Screenshots illustrating each advice type are given in Multimedia Appendix 1.

^b^ NHS Direct is a national telecare triage system providing 24-hour telephone support. We advised contacting NHS Direct in the first instance, as this service offers instant personal triage regarding appropriate next steps (eg, call ambulance, see doctor next day, etc).

The intervention was created by the research team using the LifeGuide software [31]. To ensure that the advice was safe and medically appropriate, we drew on the latest evidence-based medicine (eg, Cochrane systematic reviews, UK National Institute for Health and Clinical Excellence guidelines) and the clinical expertise of members of the research team. The content of the information provided was also informed by psychological theory. Drawing on Bandura’s Social Cognitive Theory [32], we sought to increase confidence to self-care (self-efficacy) by providing in-depth information to enhance skills and perceived capabilities for managing symptoms (particularly in the Treatment pages), and provided “vicarious learning” information about successful coping experiences of others who had used these self-care methods (eg, in clinical trials). In the Diagnostic pages we provided information on each aspect of symptoms identified by Leventhal’s model [33] as important to self-regulation of illness–that is, identity (characteristic symptomatology), cause, timeline, consequences, and possibilities for control or cure.

### Measures

Table 2 summarizes the measures used in this study, providing the full wording for items constructed for this study, and giving the reliability of multiple item scales.

**Table 2:** Final and intermediate outcome measures

Time point/target construct		Scale/item^a^	alpha^b^
**Final (4-week) follow-up**			
	Enablement	*Patient Enablement Instrument* [29]	
	Health Services Use	Three items asking whether since using the website the respondent had contacted (1) their general practitioner (or other practice staff), (2) NHS Direct or the National Pandemic Flu Service^c^, or (3) any other health care services (eg, accident and emergency)	
**Intermediate (48-hour) follow-up**			
	Satisfaction	Three items assessing satisfaction with and trust in the website advice (see Table 3)	.89
**Baseline and intermediate (48-hour) follow-up**			
	Intentions	*Intentions to consult*	.97
		I plan to go to see a doctor for my symptoms	
		I intend to go to a doctor for my symptoms	
	Self-efficacy	*Confidence to self-care*	.94
		I know what to do about my symptoms	
		I can care for my symptoms myself	
		I can cope with my symptoms without going to a doctor	
	Outcome expectancies	*Consultation necessity beliefs*	.92
		I will get better more quickly if I go to see a doctor	
		Seeing a doctor will help me to recover	
		My illness may get worse if I do not see a doctor	
		I could become very ill if I do not see a doctor	
	Illness perceptions	*Poor understanding of illness* (“coherence” subscale of Illness Perceptions Questionnaire - Revised [25])	.95
		*Emotional reactions to illness* (emotional representations’ subscale of Illness Perceptions Questionnaire – Revised [25])	.91

^a^ Full wording of items is provided for measures newly constructed for this study.

^b^ Cronbach alpha coefficient is provided for scales newly constructed for this study.

^c^ Data were collected during a period in which government advice was to contact the National Pandemic Flu Service for flu symptoms.

We assessed the primary outcomes at final (4-week) follow-up by two measures. The Patient Enablement Instrument [29] was used to measure confidence to self-manage illness; the stem was modified so that instead of asking respondents to indicate how they felt “As a result of your consultation,” they were asked to indicate how they felt “Compared with before you read the Internet Doctor webpages.” Health services usage was assessed by 3 items asking whether the respondent had contacted their general practitioner, telecare (NHS Direct), or other health care services for the symptoms they had used the website for. Predictors and intermediate outcomes were measured by scales assessing beliefs theoretically likely to predict consultation, and that the Internet Doctor was intended to influence, namely *intentions to consult* a doctor, *confidence to self-care* (ie, self-efficacy for self-management), and *consultation necessity beliefs* (ie, outcome expectancies that the illness might get worse or last longer unless the respondent consulted a doctor). Relevant illness perceptions, comprising *poor understanding of illness* and *emotional reactions to illness*, were assessed by subscales from the Revised Illness Perception Questionnaire [25], omitting the reversed items due to an unreliable pattern of responses to these items. For ease of responding, all scales were constructed from items scored from 0 (strongly disagree) to 10 (strongly agree). At baseline, additional questions recorded age, gender, and education. At the first follow-up, three additional items (see Table 3) were used as a scale measuring website satisfaction.

### Analysis

Numbers of participants completing study measures and phases varied, and so precise numbers are given for each analysis. Since many variables were somewhat skewed toward low concern about symptoms, we used conservative nonparametric tests to compare groups on the final outcome variables. We used a 2-tailed Kruskal-Wallis test for between-group comparisons in patient enablement scores, and a 2-tailed chi square test to compare numbers contacting health services.

Parametric analyses were used for the secondary analyses, as there are no satisfactory nonparametric tests for time-by-group interactions and analysis of variance (ANOVA) is robust to violations of the assumption that variables are normally distributed, unless sample sizes are small [34]. To examine change in intentions and proposed mediating variables, 2 × 2 factorial ANOVAs were carried out to test for main effects of change over time, between-group differences, and interactions between group and change. The web satisfaction items were normally distributed and so groups were compared using independent *t* tests. Independent *t* tests were also used to compare the baseline scores of those who did and did not drop out before the first follow-up.

Hierarchical linear regression was employed to examine predictors of change in intentions, pooling data across both groups. To identify bivariate predictors of change in intentions, separate regressions of each baseline and website usage predictor were carried out with intentions at follow-up as the dependent variable, controlling for baseline intentions. We then carried out a multiple regression to determine whether (1) psychological variables predicted change in intentions after controlling for relevant demographic variables, and (2) use of the Diagnostic Webpages predicted change in intentions after controlling for relevant demographic and psychological variables (ie, those with a significant bivariate relationship to change in intentions). For this regression, after controlling for baseline intentions in step 1, age was entered in step 2 (dichotomized into aged under or over 25 because of a marked skew). In step 3 we entered consultation necessity beliefs (since theory predicts these should be directly related to intentions) and in step 4 we entered illness perceptions (poor understanding of illness and emotional reactions). Finally, in step 5 we entered use of the Diagnostic Webpages. We inspected the residuals from the final regression equation to confirm that they were normally distributed.

## Results

**Figure 2:**
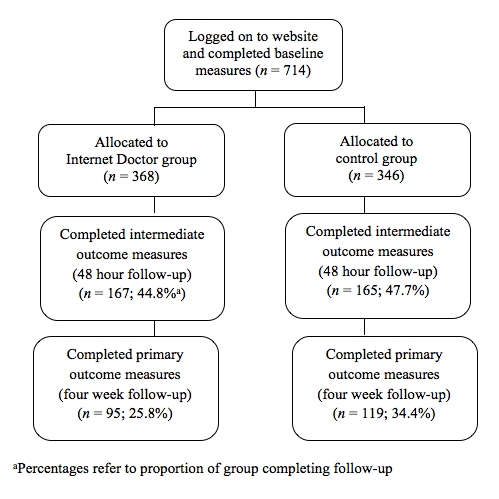
Flow of participants through the trial. ^a^Percentages refer to proportion of group completing follow-up

Baseline measures were completed by 714 people; 368 (51.5%) were randomized to the Internet Doctor website and the remainder to the static website control (see Figure 2 for flow through the study). There were 198 (27.7%) men and 516 (72.3%) women with an age range of 18 to 79, but most (440/709, 62.1%) were aged under 25. The vast majority (651, 91.2%) were completing or had completed a university degree.

### Comparison of Internet Doctor and Control Groups on Primary Outcomes at 4-Week Follow-up

Of the 214 people who completed the measures of the target outcomes at the final (4-week) follow-up, 95 (44.4%) had been assigned to the Internet Doctor group. The median patient enablement score was significantly greater in the Internet Doctor group than in the control group (median score of 3 vs 2, with an interquartile range of 0 to 5 for the whole sample, *P* = .03). Of the people in the Internet Doctor group, 11 (11.6%) had consulted their doctor or used other health care services (mainly NHS Direct) for their symptoms, compared with a substantially greater proportion (21; 17.6%) in the control group, although this difference did not approach significance in this small sample (*P* = .22).

### Comparison of Internet Doctor and Control Groups on Intermediate Outcomes at 48-Hour Follow-up

Of the 332 (46.5%) people who completed the intermediate outcomes at first follow-up, 167 (50.3%) were in the Internet Doctor group. The Internet Doctor group rated the website more positively than the control group did on all satisfaction measures (see Table 3). Comparison of those who did and did not complete the first follow-up showed that those who dropped out had significantly more negative beliefs about self-management of symptoms (*P* < .01 for all measures).

**Table 3:** Satisfaction with web-delivered advice in the Internet Doctor and control groups (n = 332)

Scale/item	Mean (SD) for each group		*P*
	Internet Doctor	Control	
Total scale (summed items divided by 3)	6.58 (1.96)	5.86 (2.27)	.002
The website gave me all the advice I needed	6.40 (2.05)	5.63 (2.51)	.002
The website was helpful to me	6.41 (2.17)	5.72 (2.51)	.007
I felt I could trust the website	6.91 (2.21)	6.25 (2.54)	.01

Intentions to consult the doctor declined between baseline and the intermediate (48-hour) follow-up; although the decline was greater in the Internet Doctor group this difference did not reach significance (see Table 4). Consultation necessity beliefs and emotional reactions to illness declined at follow-up to a similar extent in both groups. Poor understanding of illness declined in the Internet Doctor group but slightly increased in the control group, resulting in a just significant interaction between time and group effects. Self-confidence to self-care remained stable, similar and high in both groups at both time points.

**Table 4:** Intentions and attitudes at baseline and intermediate follow-up (n = 332)

	Internet Doctor group means (SD)		Control group means (SD)		*P*^a^	*P*^b^	*P*^c^
Scale	Baseline	Follow-up	Baseline	Follow-up	Time	Group	Interaction
Intention to consult doctor	2.00 (2.57)	1.66 (2.32)	1.88 (2.57)	1.82 (2.45)	.03	.93	.11
Consultation necessity beliefs	2.54 (2.25)	2.29 (2.37)	2.38 (2.23)	2.03 (2.37)	.01	.62	.61
Confidence to self-care	7.75 (2.00)	7.69 (2.08)	7.78 (1.97)	7.80 (2.01)	.84	.73	.62
Poor understanding of illness	1.86 (2.13)	1.65 (1.92)	1.64 (2.05)	1.70 (2.07)	.29	.70	.05
Emotional reactions to illness	2.36 (2.14)	2.03 (2.21)	2.40 (2.42)	2.17 (2.30)	<.001	.70	.53

^a^ Significance of main effect for time, ie, change from baseline to follow-up

^b^ Significance of main effect for between-group difference

^c^ Significance of interaction between time and group effects, ie, group difference in change from baseline

### Understanding Website Usage and its Relationship to Outcomes

The mean duration of website usage in the Internet Doctor group was 454 seconds (around 8 minutes), with a range from 24 seconds to over 52 minutes. Of the 368 people randomized to the website, 280 (76.1%) looked through the pages. Just over half (196; 53.3%) entered the Diagnostic section, a similar proportion (203; 55.2%) looked at the Treatment section, and over a quarter (104; 28.3%) looked at the Common Questions. Examination of the numbers of participants using each individual webpage revealed very diffuse usage, with virtually every page being used by at least some participants. Advice was provided for 146 symptoms, comprising runny nose in 57 (39.0%) cases, cough in 50 (34.2%) cases, sore throat in 29 (19.9%) cases, and fever in 10 (6.8%) cases. In 30.8% (45) of these cases the advice given was to contact health services.

Twenty-one people advised to contact health services completed the intermediate follow-up. There was no difference in satisfaction levels between those who were and those who were not advised to contact health services (mean 6.79, SD 2.03 and mean 6.18, SD 2.15, respectively; *P* = .21). However, intention to consult the doctor actually declined more in those advised to contact health services (*P* = .02). This was because those advised to contact health services had a higher level of intention to consult the doctor at baseline than those not advised to contact health services (mean 2.83, SD 2.98 and mean 1.88, SD 2.53, respectively), whereas at follow-up intentions were similar in both groups (mean 1.83, SD 2.59 and mean 1.73, SD 2.37, respectively).

Regression analysis (see Table 5) confirmed that reduction in intentions to consult across both groups was predicted by all the baseline measures of cognitions and illness perceptions, except for confidence to self-care. Being under 25 predicted a reduction in intentions to consult, but there were no gender differences. Use of the Diagnostic section predicted reduction in intentions, but the effect of use of the Treatment section did not quite reach significance. After controlling for the effects of age, both consultation necessity beliefs and emotional reactions to illness continued to predict reduction in intentions. After controlling for all these variables, use of the Diagnostic section remained a significant predictor of reduction in intentions to consult.

**Table 5:** Baseline and website usage predictors of intentions to consult the doctor at intermediate follow-up, controlling for intentions at baseline

Variables	Bivariate regressions^a^		Hierarchical regression^b^	
	Beta	*P*	Final beta	*P*
Baseline intentions	.78	<.001	.617	<.001
Age less than 25	.10	.003	.07	.04
Gender	.01	.79	-	-
Consultation necessity beliefs	.19	<.001	.13	.01
Confidence to self-care	-.04	.30	-	-
Poor understanding of illness	.11	.004	.05	.18
Emotional reactions to illness	.15	<.001	.11	.01
Diagnostic section used	.09	.007	.08	.02
Treatment section used	.06	.07	-	-

^a^ Intention to consult the doctor entered in step 1, then contribution of each variable examined independently.

^b^ Intention to consult the doctor entered in step 1, then variables entered in order shown, omitting those with nonsignificant bivariate relationships to intention change (see Method for details and rationale). Beta weights shown are for the last step of the equation.

## Discussion

The findings from this study suggest that tailored website advice may prove superior to simply providing written information about self-care. The Internet Doctor advice was rated as more helpful and trustworthy than the control information and resulted in higher levels of patient enablement a month later. Understanding of illness improved in the 48 hours following use of the Internet Doctor webpages, whereas there was no improvement in understanding of illness in the control group.

The shift toward weaker intentions to consult the doctor after using the website was more marked for the student-aged participants, consistent with our expectation that providing advice on self-care (in both groups) would have more influence on the intentions of those with less experience of independent self-care. As expected, reduction in intentions to consult was also predicted by prior beliefs that consultation was necessary to achieve recovery, poor understanding of illness, and greater emotional reactions to illness. This finding confirms that providing advice is likely to have most influence on the consultation rates of those who are most puzzled and distressed by their symptoms, and concerned that they may not recover without medical help. This profile matches that of patients who are more likely to consult [1, 33,35], suggesting that the advice is proving relevant to this target population.

Use of the Diagnostic section of the Internet Doctor website predicted a reduction in the strength of intentions to consult, whereas use of the Treatment section did not. This finding is not entirely surprising, since only the Diagnostic section provided specific advice about whether medical help was necessary. However, an unexpected finding was that confidence to self-care was unrelated to change in intentions to consult. Since confidence that one can carry out a behavior successfully (self-efficacy) is usually a strong predictor of behavior [21], one might expect confidence in self-care to reduce the perceived need for, and therefore intention to seek, medical help. The finding that in this case self-efficacy did not predict intentions to consult may explain why use of the Treatment section was also unrelated to consultation intentions, since this section was intended primarily to increase confidence in self-care. However, it appears that consultation is motivated more by concern about serious illness requiring medical care rather than by the desire for advice on how to relieve symptoms. Indeed, both groups already had high and stable levels of confidence in their ability to cope with these common, minor symptoms. This ceiling effect may also explain why the intervention did not produce increases in the already high levels of self-efficacy, whereas web-based interventions for more serious mental and physical conditions have been shown to increase self-efficacy [32].

Only a minority of people were advised to contact health services, a much smaller proportion than in previous studies of triage for minor symptoms [20,36]. The low rate of advice to use health services could be due to our sample of young, healthy people, who were consulting mainly for minor symptoms, but could also reflect a triage system that had a slightly higher severity threshold for recommending contacting health services. The relatively low numbers of people the system advised to contact health services is compatible with the finding that participants using the Diagnostic section were less likely to intend to consult. However, an unpredicted finding was that intentions to consult the doctor actually declined more in those who were advised to contact health services. This might simply be because they had more severe symptoms at baseline, which then abated during the 48 hours before follow-up. Since advice to contact health services was accompanied by an explanation of which symptoms were of concern, an alternative possibility is that participants used this information to monitor these symptoms for improvement after using the Internet Doctor.

### Strengths and Limitations of the Study

This study had a number of strengths as a direct test of the effects of tailored advice in the context of self-management of minor symptoms: in particular, a direct comparison with the best existing nontailored patient information, and detailed analysis of reliable, theory-based measures of relevant beliefs and perceptions. However, the findings cannot be considered definitive. The sample size was too small to reliably detect group differences in consultation rates, and reported consultation rates were not objectively verified. Future research should evaluate the effects on recorded consultations in a much larger sample, following all the usual conventions for a full trial.

While our study design provided a strong test of the efficacy of tailoring information, it did not permit evaluation of the effectiveness of the website for reducing consultation rates, since the control group was given nontailored advice that was previously shown to be effective in reducing consultation rates. It is encouraging that a reduction in intentions to consult, consultation necessity beliefs, and emotional reactions was seen in both groups after using the website. However, a further trial is needed, including comparison with a control group who are not given access to any triage advice, in order to determine to what extent reductions in consultations intentions are due to receiving web-based advice.

There was substantial dropout before follow-up, which is a common problem in internet studies with volunteer samples [37-39]. Those who dropped out had less confidence to self-manage their symptoms, suggesting that the reductions in concern about symptoms seen in those who were followed up might not have been observed in those who dropped out. Overall satisfaction levels even in those who completed the study were only mildly positive; findings from a qualitative study of responses to the Internet Doctor [40] suggest that this may be because the somewhat restricted computer-tailored advice is often perceived as inferior to the detailed personal advice that a health professional can provide. Further research and development is required in order to try to determine whether it is possible to achieve higher levels of satisfaction, and whether these might attenuate attrition [39]. This is particularly important, as our sample is likely to have had more positive attitudes toward web-based advice than might be found in the general population. In addition to being volunteers, our sample mainly comprised students, and web-based advice may prove less appealing and effective in older and less well-educated populations, since they tend to have lower levels of self-efficacy both for web usage and for self-management of health [10,41]. In addition, women were substantially overrepresented in our sample (although the proportion was similar to the take-up observed in an observational study of providing digital triage for a student population [20]). There is evidence that women tend to have a more positive attitude than men toward self-management of health [42]. For these reasons, future research should be carried out in a more representative population sample.

### Conclusions

Our findings provide initial evidence that tailored web-based advice could help patients self-manage minor symptoms to a greater extent. Effect sizes on consultation rates were modest, which is consistent with previous research suggesting that often information may be obtained from the Internet in order to supplement rather than replace consultations with doctors [12,41,43]. Nevertheless, if replicated, these effect sizes would be potentially very valuable if the intervention were rolled out widely. Consequently, these findings constitute a sound foundation and rationale for future research. In particular, our study provides the evidence required to justify carrying out much larger trials in representative population samples comparing tailored web-based advice with routine care, in order to obtain a definitive evaluation of the effects on self-management and health service use.

## Multimedia Appendix

Illustrative screen shots of the Internet Doctor

## Abbreviations

ANOVA: analysis of variance
RCT: randomized controlled trial

## References

[1] Rogers A, Hassell K, Nicolaas G (1999). Demanding Patients: Analysis of the Use of Primary Care.

[2] McCormick A, Fleming D, Charlton J (1995). Morbidity statistics from general practice: fourth national study 1991-1992.

[3] Department of Health.

[4] Santana S, Lausen B, Bujnowska-Fedak M, Chronaki C, Kummervold PE, Rasmussen J, Sorensen T (2010). Online communication between doctors and patients in Europe: status and perspectives. J Med Internet Res.

[5] Hardey M (1999). Doctor in the house: the Internet as a source of lay health knowledge and the challenge to expertise. Sociol Health Illn.

[6] Korp P (2006). Health on the Internet: implications for health promotion. Health Educ Res.

[7] McCarthy K, Prentice P (2006). Commissioning health education in primary care. BMJ.

[8] Department of Health.

[9] Little P, Somerville J, Williamson I, Warner G, Moore M, Wiles R, George S, Smith A, Peveler R (2001). Randomised controlled trial of self management leaflets and booklets for minor illness provided by post. BMJ.

[10] Rogers A, Mead N (2004). More than technology and access: primary care patients' views on the use and non-use of health information in the Internet age. Health Soc Care Community.

[11] Henwood F, Wyatt S, Hart A, Smith J (2003). 'Ignorance is bliss sometimes': constraints on the emergence of the 'informed patient' in the changing landscapes of health information. Sociol Health Illn.

[12] Sommerhalder K, Abraham A, Zufferey MC, Barth J, Abel T (2009). Internet information and medical consultations: experiences from patients' and physicians' perspectives. Patient Educ Couns.

[13] Vingilis E, Brown U, Koeppen R, Hennen B, Bass M, Peyton K, Downe J, Stewart M (1998). Evaluation of a cold/flu self-care public education campaign. Health Educ Res.

[14] Coulter A, Ellins J (2007). Effectiveness of strategies for informing, educating, and involving patients. BMJ.

[15] Macfarlane JT, Holmes WF, Macfarlane RM (1997). Reducing reconsultations for acute lower respiratory tract illness with an information leaflet: a randomized controlled study of patients in primary care. Br J Gen Pract.

[16] Morrell DC, Avery AJ, Watkins CJ (1980). Management of minor illness. Br Med J.

[17] Kreuter M (2000). Tailoring health messages: customizing communication with computer technology.

[18] Williams P, Huntington P, Nicholas D (2003). Health information on the Internet: a qualitative study of NHS Direct Online users. Aslib Proc.

[19] Nijland N, van Gemert-Pijnen J, Boer H, Steehouder MF, Seydel ER (2008). Evaluation of internet-based technology for supporting self-care: problems encountered by patients and caregivers when using self-care applications. J Med Internet Res.

[20] Sole ML, Stuart PL, Deichen M (2006). Web-based triage in a college health setting. J Am Coll Health.

[21] Bandura A (1997). Self-efficacy: the exercise of control.

[22] Maddux JE, Brawley L, Boykin A, Maddux  JE (1995). Self-efficacy and healthy behavior: prevention, promotion, and detection. Self-Efficacy, Adaptation, and Adjustment: Theory, Research, and Application.

[23] Bass SB, Ruzek SB, Gordon TF, Fleisher L, McKeown-Conn N, Moore D (2006). Relationship of Internet health information use with patient behavior and self-efficacy: experiences of newly diagnosed cancer patients who contact the National Cancer Institute's Cancer Information Service. J Health Commun.

[24] Leventhal HA, Brissette I, Leventhal EA, Cameron LD, Leventhal  HA (2003). The common-sense model of self-regulation of health and illness. The self-regulation of health and illness behaviour.

[25] Moss-Morris R, Weinman J, Petrie KJ, Horne R, Cameron LD, Buick D (2002). The Revised Illness Perception Questionnaire (IPQ-R). Psychol Health.

[26] Ajzen I (1991). The theory of planned behavior. Organizational Behavior and Human Decision Processes.

[27] Nijland N, Cranen K, Boer H, van Gemert-Pijnen JE, Seydel ER (2010). Patient use and compliance with medical advice delivered by a web-based triage system in primary care. J Telemed Telecare.

[28] Craig P, Dieppe P, Macintyre S, Michie S, Nazareth I, Petticrew M (2008). Developing and evaluating complex interventions: the new Medical Research Council guidance. BMJ.

[29] Howie JG, Heaney DJ, Maxwell M, Walker JJ (1998). A comparison of a Patient Enablement Instrument (PEI) against two established satisfaction scales as an outcome measure of primary care consultations. Fam Pract.

[30] Yardley L, Morrison LG, Andreou P, Joseph J, Little P (2010). Understanding reactions to an internet-delivered health-care intervention: accommodating user preferences for information provision. BMC Med Inform Decis Mak.

[31] Yang Y, Osmond A, Chen X, Weal M, Wills G, de Roure D, Joseph J, Yardley L (2009). Supporting the running and analysis of trials of web-based behavioural interventions: the LifeGuide.

[32] Samoocha D, Bruinvels DJ, Elbers NA, Anema JR, van der Beek AJ (2010). Effectiveness of web-based interventions on patient empowerment: a systematic review and meta-analysis. J Med Internet Res.

[33] Cameron L, Leventhal EA, Leventhal H (1993). Symptom representations and affect as determinants of care seeking in a community-dwelling, adult sample population. Health Psychol.

[34] StatSoft Inc (2010). Electronic Statistics Textbook.

[35] Cornford CS (1998). Why patients consult when they cough: a comparison of consulting and non-consulting patients. Br J Gen Pract.

[36] Nijland N, Cranen K, Verlinden SFF, Kelders SM, Boer H (2009). Computer generated self-care advice via web-based triage of complaints in primary care.

[37] Kraft P, Yardley L (2009). Current issues and new directions in Psychology and Health: What is the future of digital interventions for health behaviour change?. Psychol Health.

[38] Suggs LS, Cowdery JE, Carroll JB (2006). Tailored program evaluation: Past, present, future. Eval Program Plann.

[39] Eysenbach G (2005). The law of attrition. J Med Internet Res.

[40] Morrison L, Joseph J, Andreou P, Yardley L (2009). Application of the LifeGuide: a think-aloud study of users' experiences of the 'Internet Doctor'.

[41] Baker L, Wagner TH, Singer S, Bundorf MK (2003). Use of the Internet and e-mail for health care information: results from a national survey. JAMA.

[42] Say R, Murtagh M, Thomson R (2006). Patients' preference for involvement in medical decision making: a narrative review. Patient Educ Couns.

[43] Lemire M, Sicotte C, Paré G (2008). Internet use and the logics of personal empowerment in health. Health Policy.

