# Supplementary figures and images for: Evaluation of a Web-based Intervention Providing Tailored Advice for Self-management of Minor Respiratory Symptoms: Exploratory Randomized Controlled Trial
Source: J Med Internet Res. 2010 Dec 15;12(4):e66. doi: 10.2196/jmir.1599 (PMC3056528; doi:10.2196/jmir.1599)

## Slide 1
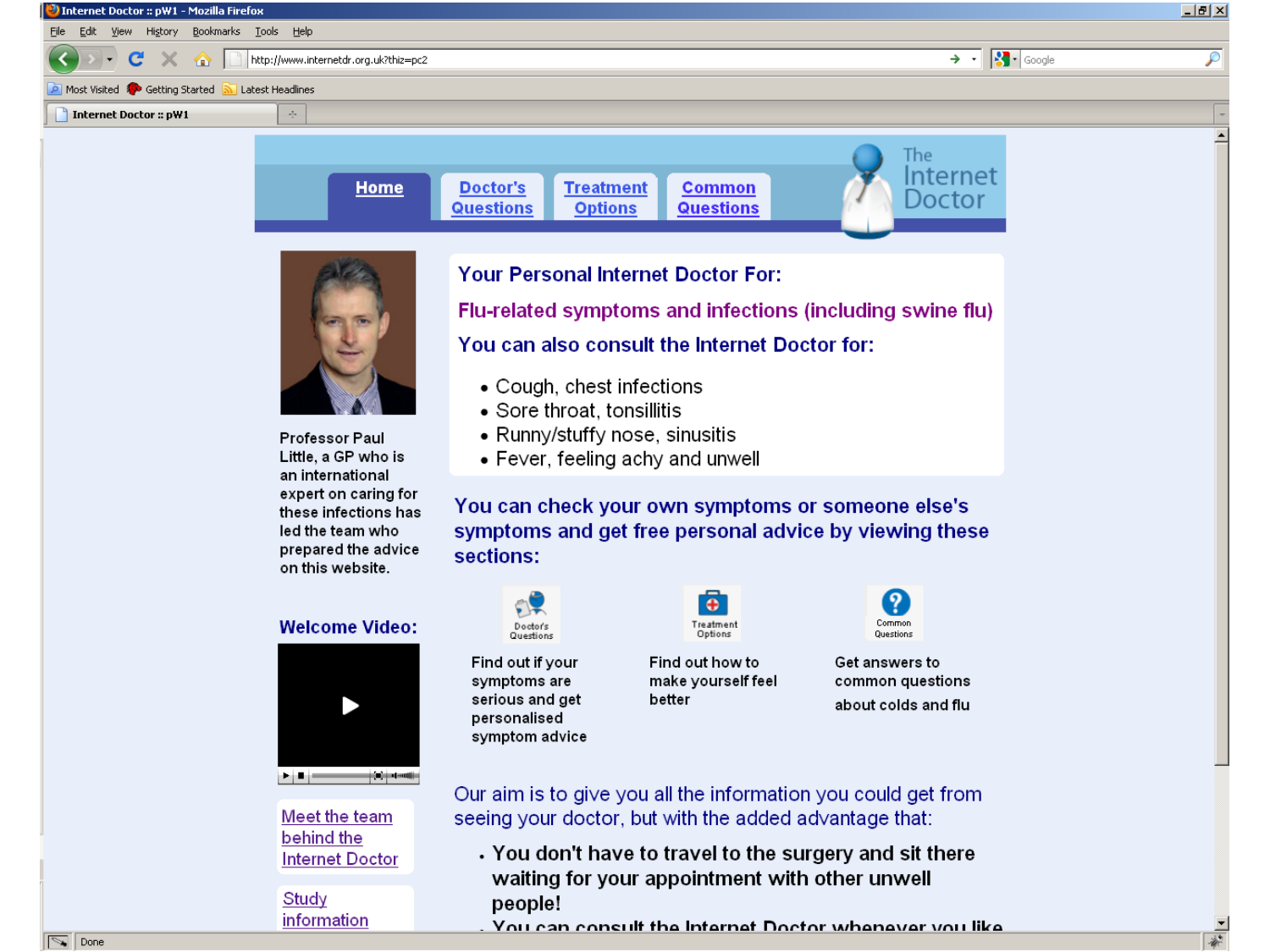

## Slide 2
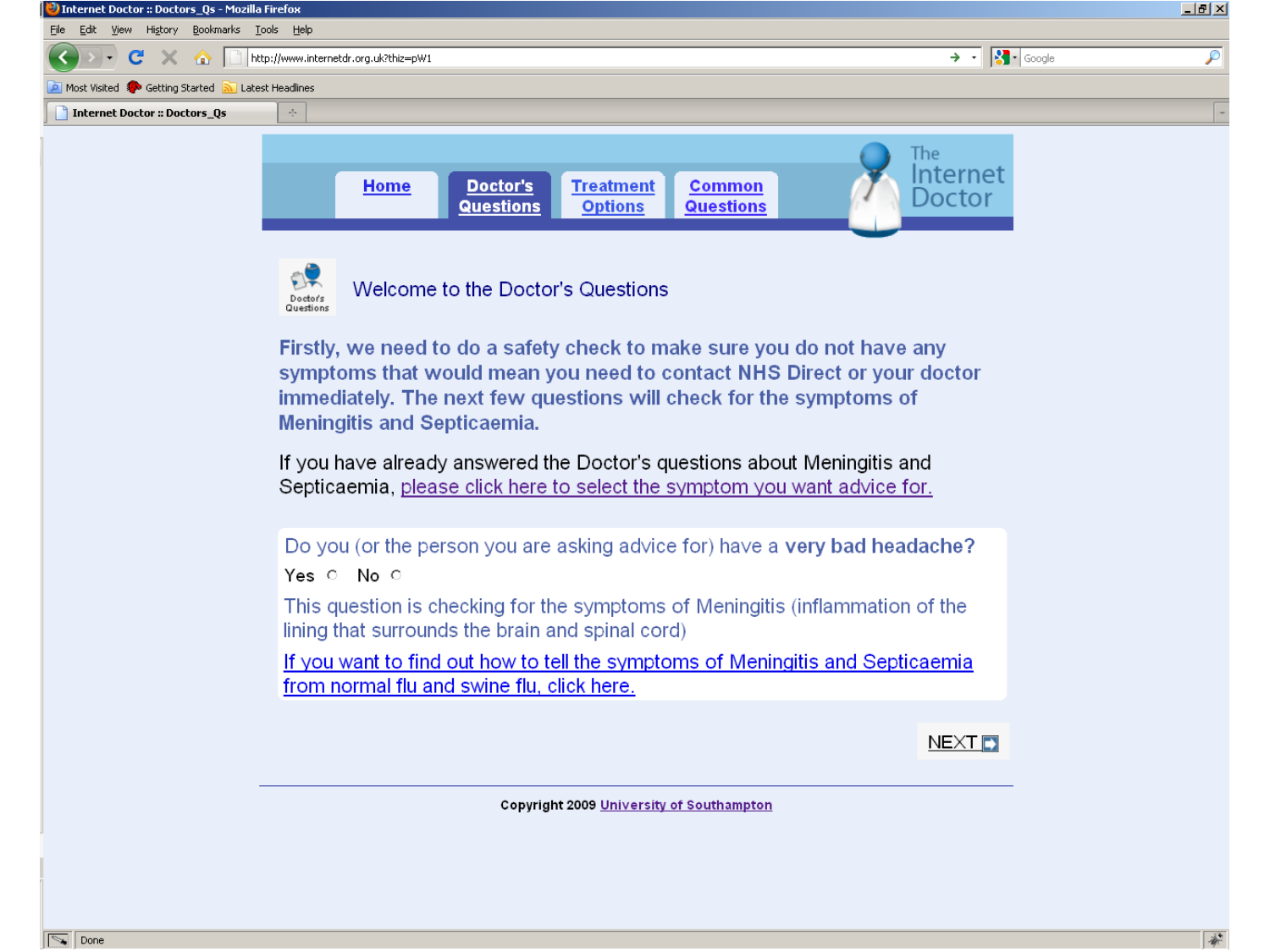

## Slide 3
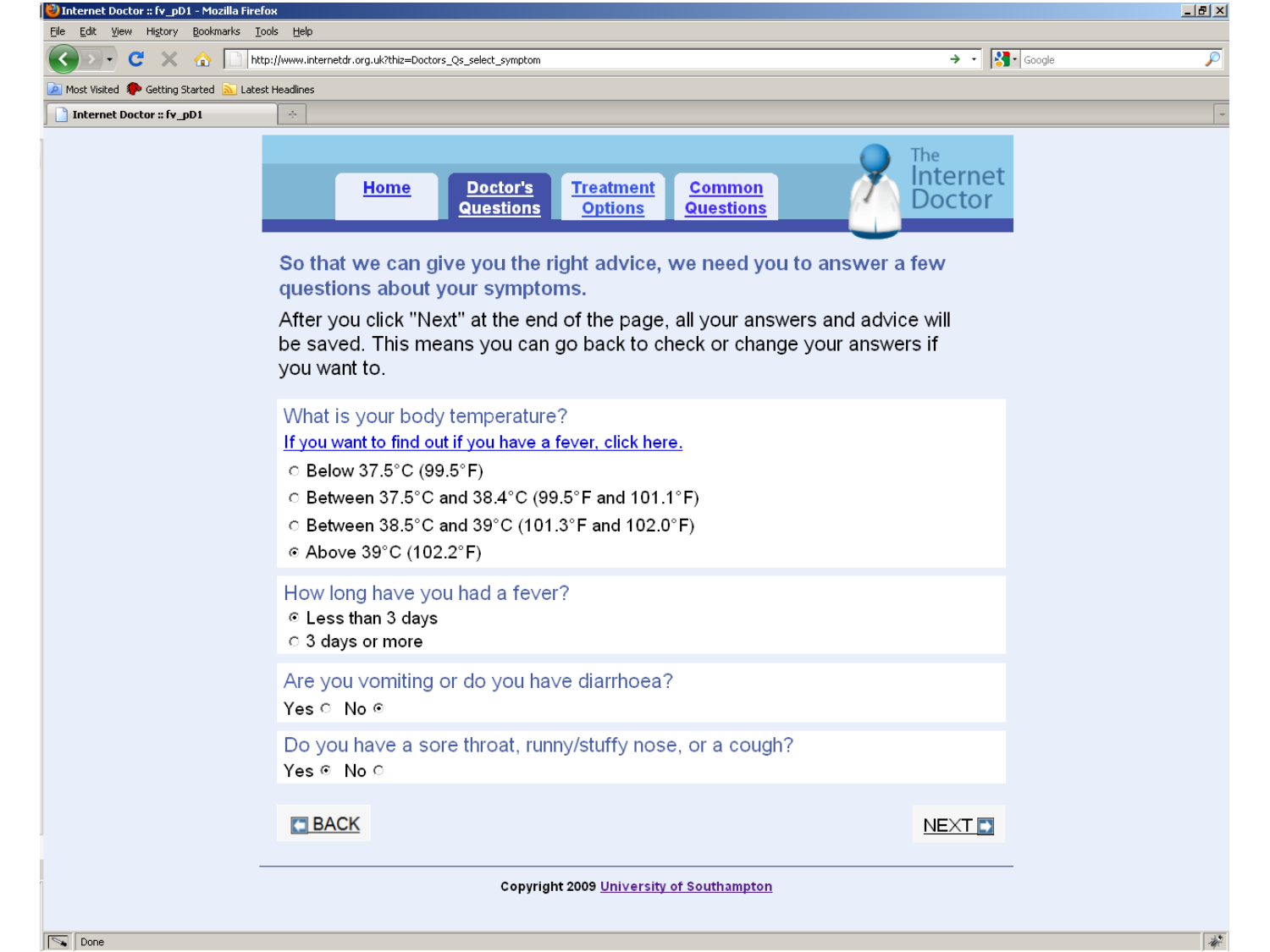

## Slide 4
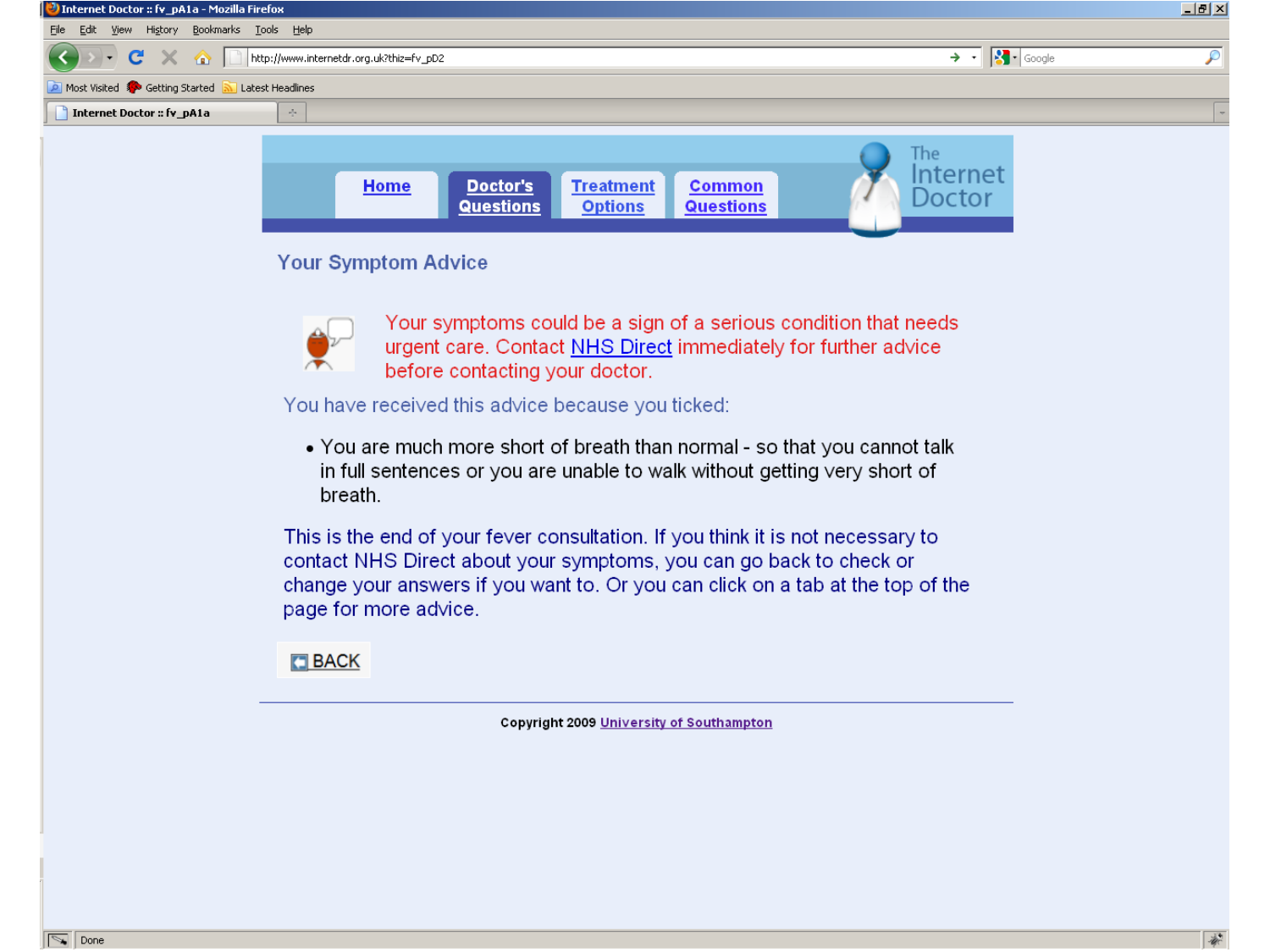

## Slide 5
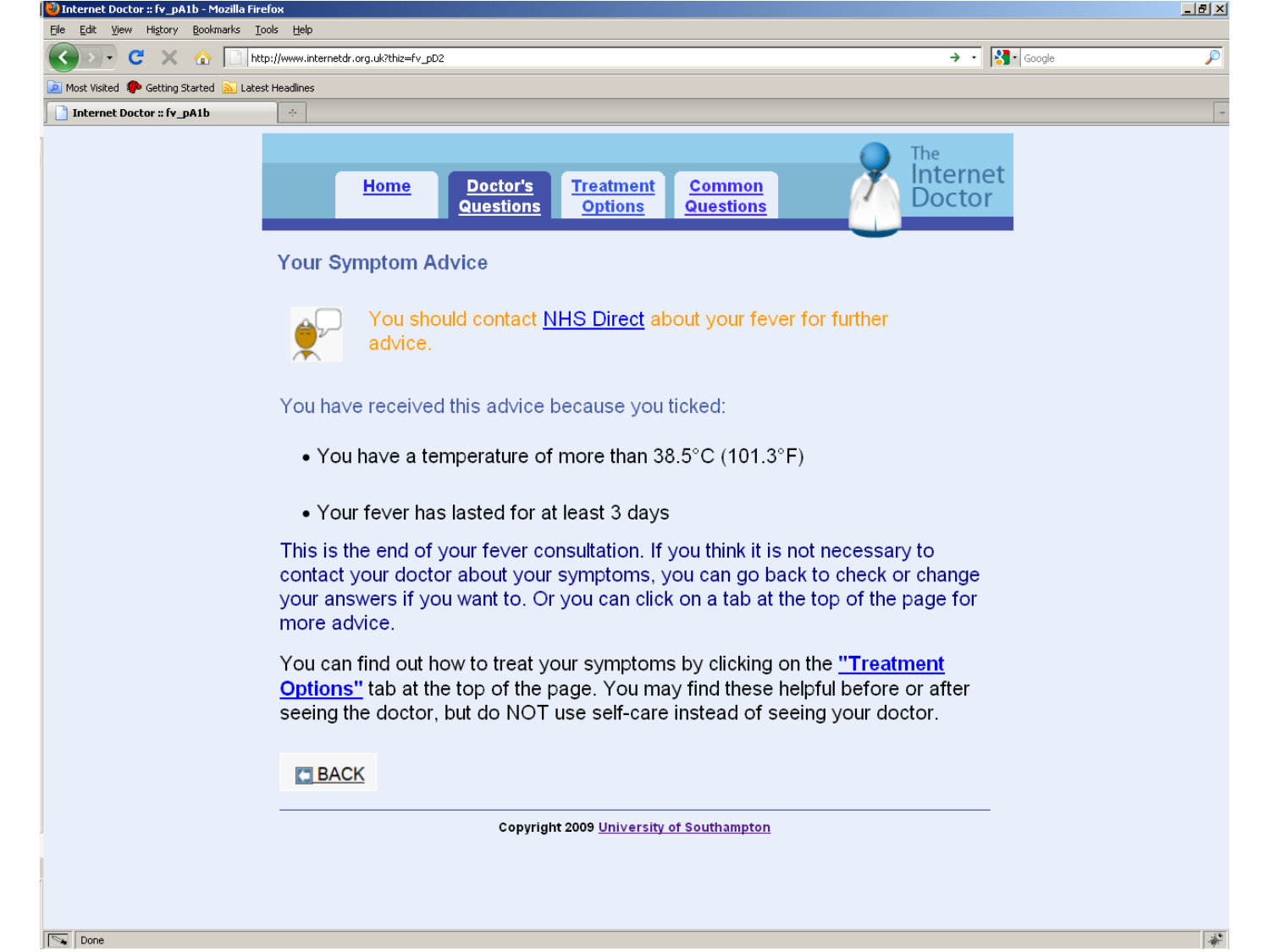

## Slide 6
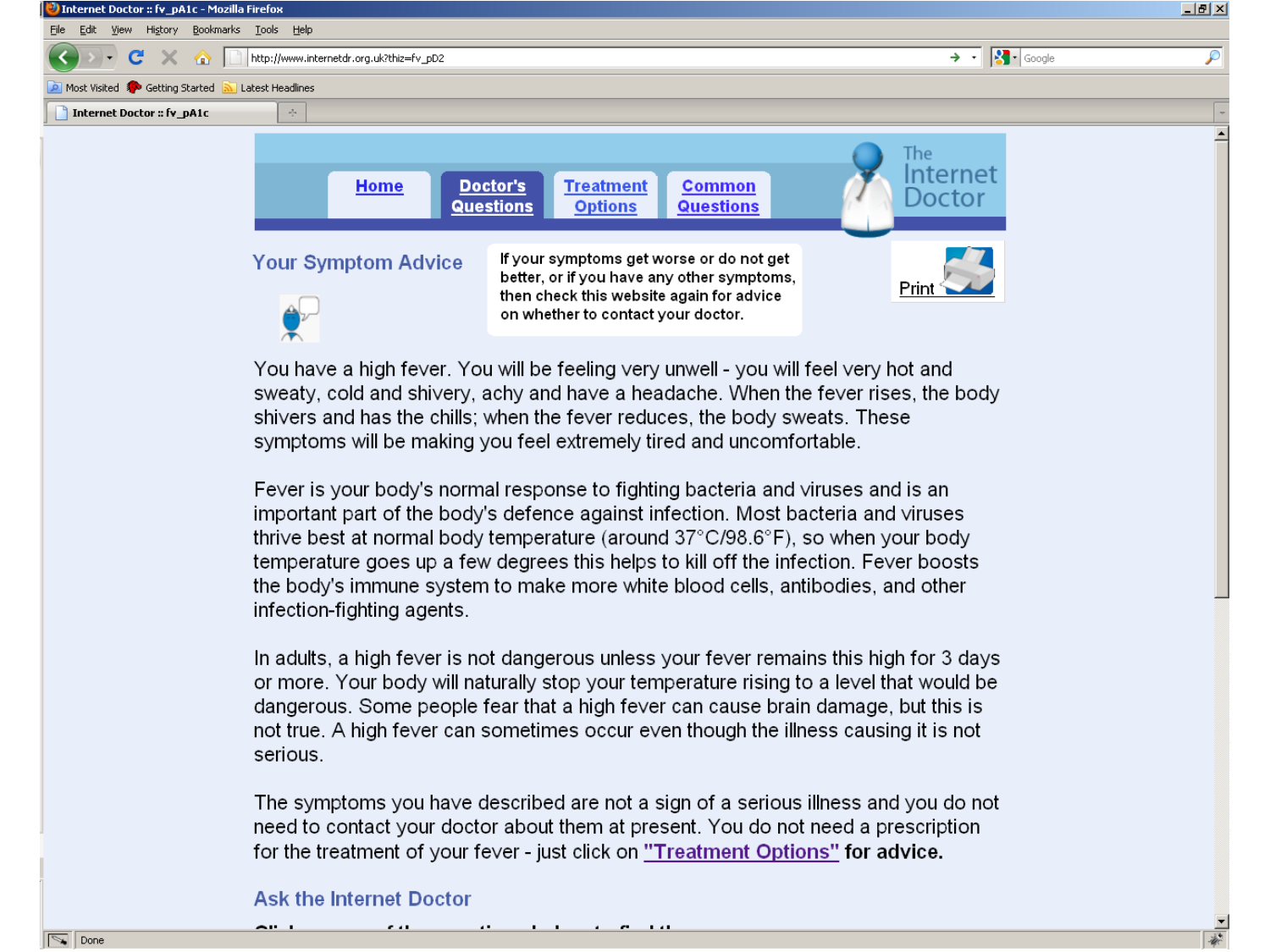

## Slide 7
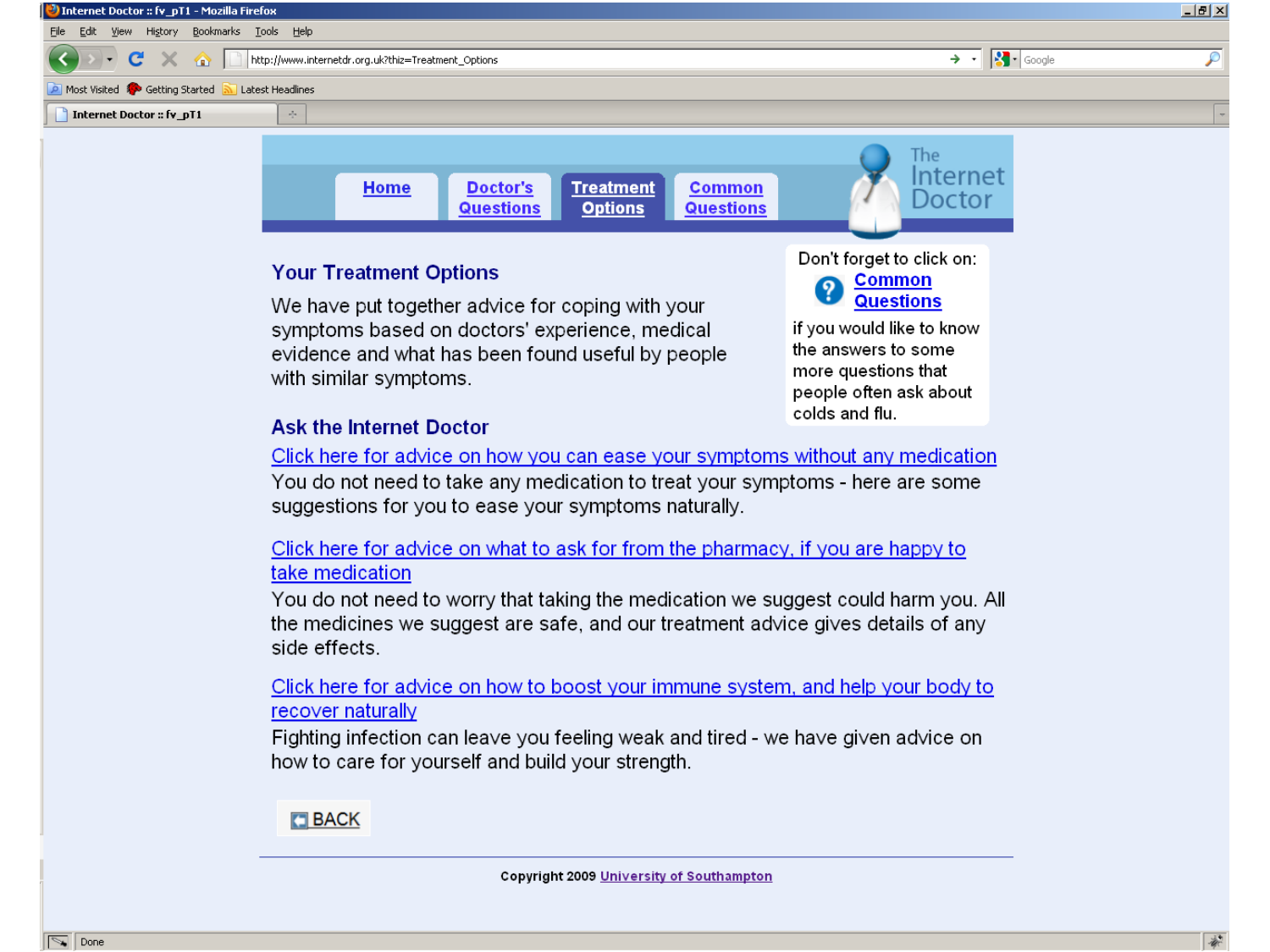

## Slide 8
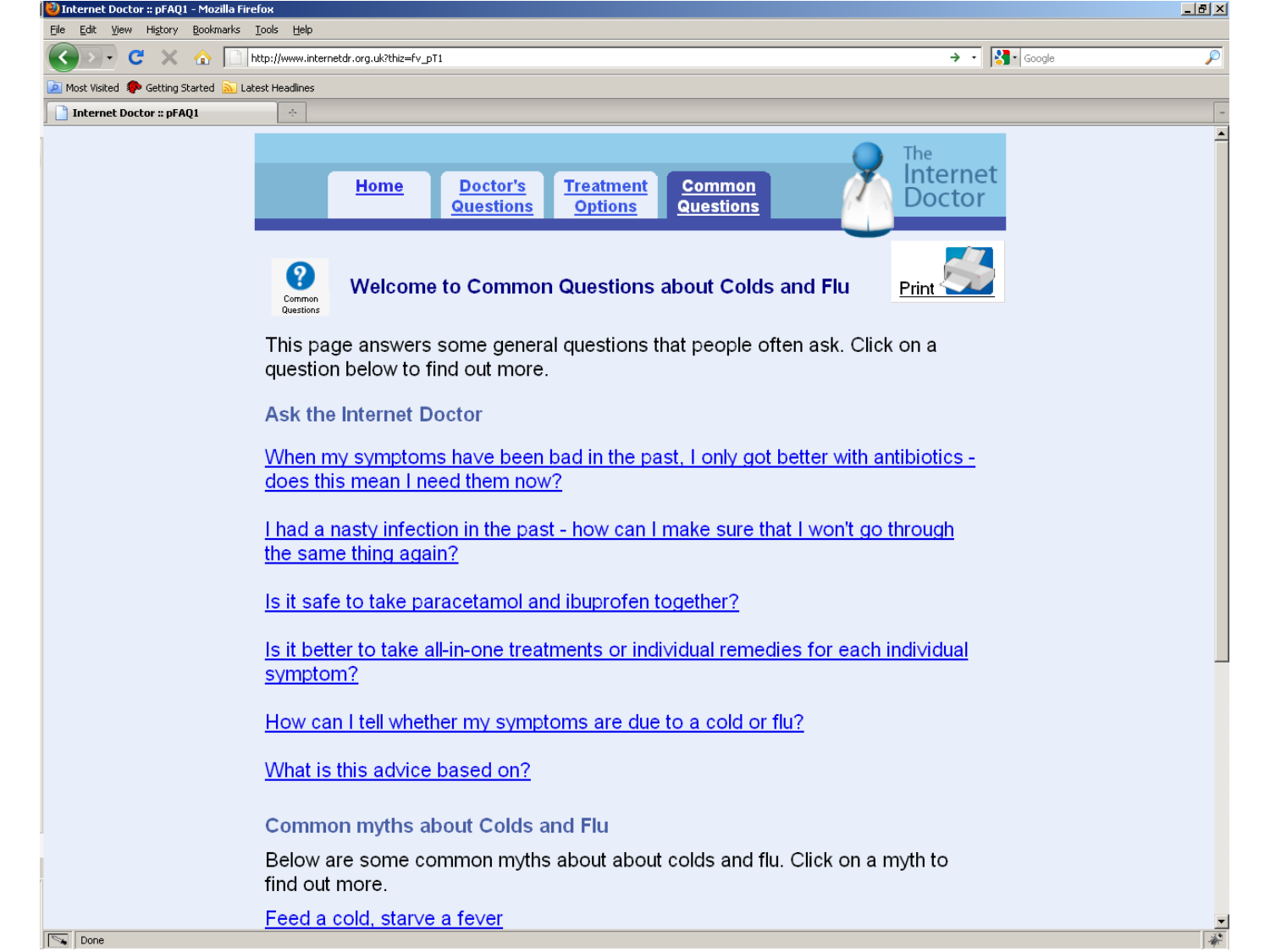

Supplement: Supplementary file 1 [file jmir_v12i4e66_app1.ppt]
